# Operative Treatment of Tuberculous Glands in the Neck

**Published:** 1903-11-28

**Authors:** 


### OPERATIVE TREATMENT OF TUBERCULOUS GLANDS IN THE NECK.

Mr. W. G. Sutcliffe[1] emphasises the necessity, when performing an operation for the removal of tuberculous glands in the neck, of completely clearing out the glands, however deeply situated, so as to avoid the recurrences which so often follow incomplete operations. Another important point is to plan the skin incision in such a way that the resulting scar shall be as inconspicuous as possible. No routine incision or operation can be laid down, because the groups of glands affected vary so much in size and situation in different cases. Mr. Sutcliffe points out that the old incision along the anterior border of the sterno-mastoid leaves a most unsightly scar, especially if the incision be a long one. It is better to make use of Kocher's suggestion whenever it is possible, and place the incision in one of the natural sulci of the neck. There are two such grooves in most necks, running obliquely across from below the lobe of the ear to the thyro-hyoid region. Where the greater part of the mass is in the posterior triangle, an additional longitudinal incision may be made from the posterior edge of the swelling downwards and obliquely forwards to the middle of the posterior border of the sterno-mastoid. In very extensive cases, where most of the glands in the neck are affected, the scar that shows the least is to be made by turning a flap forwards and downwards. The upper of the incisions which marks out the flap is made in one of the transverse sulci mentioned above.

If the patient has already had a previous operation performed, the scar of the old operation should be utilised if possible. When sinuses are present their orifices should be included in an eliptical incision which is made so as to produce a transverse scar. Any damaged skin in connection with superficial abscesses should be removed with a free hand, it being possible in nearly all cases to sew up the wound after the operation has been completed.

Whatever incision be used, attention must be paid to the internal jugular vein and the spinal accessory nerve. Mr. Sutcliffe is of opinion that it is very rarely needful to interfere with the internal jugular vein. There is a thick layer of fascia constituting the carotid sheath between the glands and the vein, and although the glands may be firmly adherent to the sheath, the sheath itself usually strips off from the vein readily enough.

Great care must be taken to avoid division of the spinal accessory nerve. If this accident happen, the drooping of the shoulder which results is a marked deformity, while considerable loss of power ensues, and, if the patient be a child, lateral curvature of the spine is nearly certain to follow.

[1] Lancet, Nov. 14.

